# Impaired Control of Epstein–Barr Virus Infection in B-Cell Expansion with NF-κB and T-Cell Anergy Disease

**DOI:** 10.3389/fimmu.2018.00198

**Published:** 2018-02-08

**Authors:** Swadhinya Arjunaraja, Pamela Angelus, Helen C. Su, Andrew L. Snow

**Affiliations:** ^1^Department of Pharmacology and Molecular Therapeutics, Uniformed Services University of the Health Sciences, Bethesda, MD, United States; ^2^Laboratory of Clinical Immunology and Microbiology, National Institute of Allergy and Infectious Diseases, National Institutes of Health, Bethesda, MD, United States; ^3^Clinical Research Directorate/Clinical Monitoring Research Program, Leidos Biomedical Research, Inc., National Cancer Institute at Frederick, Frederick, MD, United States

**Keywords:** Epstein–Barr virus, B-cell expansion with NF-κB and T-cell anergy, CARD11, NF-κB, primary immune deficiency

## Abstract

*B*-cell *e*xpansion with *N*F-κB and *T*-cell *a*nergy (BENTA) disease is a B-cell-specific lymphoproliferative disorder caused by germline gain-of-function mutations in *CARD11*. These mutations force the CARD11 scaffold into an open conformation capable of stimulating constitutive NF-κB activation in lymphocytes, without requiring antigen receptor engagement. Many BENTA patients also suffer from recurrent infections, with 7 out of 16 patients exhibiting chronic, low-grade Epstein–Barr virus (EBV) viremia. In this mini-review, we discuss EBV infection in the pathogenesis and clinical management of BENTA disease, and speculate on mechanisms that could explain inadequate control of viral infection in BENTA patients.

## Introduction

Epstein–Barr virus (EBV) is a ubiquitous human herpesvirus that establishes life-long infection in ~90% of individuals (1). Primary EBV infection in childhood is usually asymptomatic in immunocompetent hosts, while acquisition of EBV during adolescence can result in infectious mononucleosis (IM) that usually resolves within days to weeks. However, EBV infections can also trigger lymphoproliferative disease, lymphoma, fulminant infectious mononucleosis (FIM), and/or hemophagocytic lymphohistiocytosis (HLH) in genetically or iatrogenically immunocompromised patients (2–4). These conditions clearly suggest that EBV has co-evolved with its host under constant immune surveillance to ensure that virus–host homeostasis is maintained (5, 6).

The transmission, circulation, and persistence of EBV in the human host have been reviewed extensively (1, 4, 7–9). EBV initially establishes lytic infection in both B lymphocytes and epithelial cells of the oropharynx. The EBV lytic gene program ensures both viral replication and evasion from early detection by either natural killer (NK) cells or CD8^+^ T cells (10). EBV then switches to a latent infection program that expands the pool of infected B cells considerably (11). This is achieved in part through the expression of key latent membrane proteins 1 and 2A, which mimic constitutive CD40 and B cell receptor signaling, respectively. Evidence suggests that this “growth” program of latency (i.e., latency III) provides the proliferative and pro-survival signals necessary to drive the EBV-infected B cell through a germinal center-like reaction and eventually into the memory B cell pool, all without requiring cognate antigen recognition. At the same time, latent proteins such as LMP2 and EBNA3A/B/C contain potent immunodominant class I MHC peptide epitopes for CD8^+^ T cell recognition, ensuring the destruction of most EBV-infected B cells at this stage (3). CD8^+^ T (and NK) cell-mediated killing is also aided by the robust upregulation of ligands for both NKG2D and signaling lymphocyte activation molecule (SLAM) family receptors on EBV-infected B cells, which participate in signaling for cytotoxic functions (see below) (5). Surviving EBV^+^ memory B cells remain quiescently infected without expressing viral antigens (latency 0) for the lifetime of the individual, with occasional rounds of viral reactivation thought to occur as these cells traffic back through the oropharynx (12). EBV-specific cellular immunity maintains tight control throughout these cycles (5).

In this manner, EBV has evolved an elegant strategy for ensuring initial colonization followed by persistent, benign infection in the immunocompetent host, in which a sizable portion of the memory CD8^+^ T cell pool (~2–5%) is dedicated to maintaining EBV-specific immunosurveillance (3, 13). Indeed, cytolytic killing of EBV-infected B cells by effector CD8^+^ T cells occurs during acute IM, comprising up to 50% of the CD8^+^ T cell compartment (4, 14). EBV-specific CD4^+^ T cells are also important for robust CD8^+^ cellular immunity and can also participate directly in cytotoxic killing of infected B cells (15, 16). During asymptomatic infection, NK cells help to restrict viral load by inhibiting their replication and can reduce the likelihood of EBV transformation of B cells (10, 17, 18). Furthermore, other innate effectors such as invariant natural killer T cells are also known to play a role in killing of infected B cells and can limit EBV transformation of B cells *in vitro* (19).

The advent of next-generation sequencing technology has enabled us to characterize primary immune deficiency (PID) states in humans caused by mutations in single immune-related genes that predispose them to certain pathogens. Indeed, several PIDs have now been recognized for their specific susceptibility to uncontrolled EBV infection and associated disease, sometimes referred to collectively as “EBV-opathies” (5, 20–22). In this review, we focus our attention on the incidence and severity of EBV infection in a recently characterized PID known as *B*-cell *e*xpansion with *N*F-κB and *T*-cell *a*nergy (BENTA). Mechanistic insights into possible immunological shortcomings surrounding EBV infections in BENTA patients are provided below.

## Benta Disease

Our group discovered a B-cell-specific lymphoproliferative congenital human disorder termed BENTA (23). BENTA disease is caused by heterozygous, germline-encoded gain-of-function mutations in the gene *CARD11*, which encodes a lymphocyte-specific scaffold protein (CARD11) also known as CARMA1. The CARD11 protein bridges the antigen receptor ligation in B or T cells with multiple downstream signaling pathways such as canonical NF-κB, c-Jun N-terminal kinase (JNK), and mechanistic target of rapamycin (mTOR) (24–26). Subsequent to antigen receptor ligation in lymphocytes, CARD11 is phosphorylated to facilitate BCL10 and MALT1 binding to form the CARD11–BCL10–MALT1 (CBM) complex, which further nucleates the dynamic signalosomes that activate inhibitor of κB kinase (IKK) and culminate in NF-κB translocation into the nucleus (27–30) to activate the canonical NF-κB pathway. The NF-κB family of transcription factors is critical for the induction of genes involved in cell survival, proliferation, and immune effector functions (31). GOF mutations in CARD11 render the protein in an open, active state irrespective of antigen receptor engagement, resulting in constitutive NF-κB activation (31–33).

To date, 16 different BENTA patients have been identified and definitively diagnosed, with five distinct *CARD11* mutations. Polyclonal B cell lymphocytosis in early childhood is a hallmark of BENTA disease, often accompanied by splenomegaly and lymphadenopathy (23, 34–40). Immunologic phenotyping reveals the remarkable accumulation of both CD10^+^CD24^hi^CD38^hi^ transitional and IgM^+^IgD^+^ mature naïve polyclonal B cells, even though T cell numbers frequently fall within the normal range (Table 1). Many BENTA patients also present with several signs of primary immunodeficiency despite the absence of any autoimmune disease symptoms. Recurrent ear and sinopulmonary infections are common in all patients, with other opportunistic viral infections such as molluscum contagiosum, BK virus, and EBV observed in some patients. In most patients, inadequate antibody responses against T-cell independent pneumococcal and meningococcal polysaccharide-based vaccines are noted. Some patients also show poor responses to T-cell-dependent vaccines such as Varicella Zoster virus and measles. Poor humoral immune responses in these patients are also reflected in very low frequencies of circulating class-switched and memory B cells, as well as low levels of IgM and IgA in the serum. Impaired humoral immunity in BENTA is evidenced by intrinsic defects in plasma cell differentiation and antibody secretion upon stimulation of naïve patient B cells *in vitro*, despite normal proliferation and enhanced survival (41). The hyporesponsiveness of BENTA patient T cells to *in vitro* stimulation, including poor proliferation and reduced IL-2 secretion, may also contribute to defective class-switched Ab responses (23, 35).

**Table 1:** Phenotypic analysis of BENTA patients.

	Age^b^	EBV VCA-IgG	EBV LOAD	CD8^+^ T		CD4/CD8 ratio	NK		CD3^+^ NKT		CD19^+^ B	
			Log10 IU/ml	%	#/µl	Ratio	%	#/µl	%	#/µl	%	#/µl
Healthy control ref ranges	**>18 years**	Neg	UD	11.2–34.8	178–853	1.17–5.17	6.2–34.6	126–729	2.2–12.4	29–299	3–19	59–329
	**6**–**18 years**	Neg	UD	18–35	330–1,100	1.34–1.72	3–22	70–480	0.49–15^c^	12–350^c^	6–27	110–860
**BENTA patients**												
***G123S***												
**P4**	**11**	**Pos**	**3.35**	**19**	**868**	0.51	**4.9**	**224**	**2.7**	**123**	62.2	2,843
**P7**	**43**	**Pos**	**UD**	2	**300**	**5**	**ND**	**ND**	**ND**	**ND**	52	7,900
**P14**	**20**	**Pos**	**4.6**	3.3	**200**	**1.5**	**ND**	**ND**	Low	Low	83	5,000
***G123D***												
**P6**	**15**	**Pos**	**2.07**^a^	3.3	2,853	0.64	**3.5**	3,026	**1.6**	1,383	89.4	77,286
***H234L Δ235-8***												
P10	80	ND	ND	ND	ND	ND	ND	ND	ND	ND	ND	ND
**P11**	**57**	**Pos**	**3.13**	**31.7**	**970**	1.11	**8.9**	**272**	**10**	**306**	20	612
**P12**	**32**	**Pos**	**4.45**	**16.3**	**535**	**2.48**	**6.5**	**213**	**6**	**197**	32.5	1,066
P13	6	ND	Neg	13.7	507	2.55	4.1	152	3.6	133	43.3	1,602
***C49Y***												
P5	21	ND	Neg	10.7	322	1.8	2	60	2.2	66	66.4	1,938
P8	53	ND	Neg	8	152	3.37	10	190	ND	ND	48	912
P9	20	ND	Neg	11	418	2.6	6	228	ND	ND	50	1,900
**P15**	**43**	**Pos**	**2.07**	**14.6**	**298**	**1.28**	4.7	96	1.6	**33**	56	1,142
**P16**	**16**	**Pos**	**3.13**	**20.7**	**271**	**2.12**	**3.8**	50	**2.9**	**38**	21.3	**279**
***E134G***												
P1	55	Neg	Neg	34.1	1,449	1.09	5.6	238	15.5	659	18.9	803
P2	13	Neg	Neg	10.2	585	1.67	4.1	235	1.6	92	65.4	3,754
P3	11	Neg	Neg	10.6	409	1.99	3.6	139	2.1	81	61.9	2,389

*Values in blue and red color indicate lower and higher range, respectively, compared with adult (>18 years) or pediatric (6–18 years) healthy control range (42, 43) as specified in the subheader column. Adult ranges are derived from the NIH Clinical Center (Department of Laboratory Medicine). EBV seropositive carriers are in bold. Several patients have been described in published reports, including P1–P4 (23, 44), P6 (35), P7 (38), P5, P8, and P9 (36), P14 (37), and P16 (36). Other patients (P10–P13, P15) have been evaluated at the NIH Clinical Center but have not been published to date*.

*ND, not determined; UD, undetectable; Pos, positive; Neg, negative; EBV, Epstein–Barr virus; NK, natural killer; BENTA, B-cell expansion with NF-κB and T-cell anergy*.

*^a^Measurement taken while P6 was 14 years old, but for reasons unknown his EBV load was undetectable at age 15*.

*^b^Patient’s age at the time of measurement*.

*^c^Measurement range for 5- to 16-year-old healthy controls (45)*.

Eight out of 16 patients are seropositive for EBV (Table 1). While EBV viral load is generally undetectable in healthy carriers, almost all BENTA patients (7/8) exposed to EBV are demonstrably viremic as measured by their DNA copy number (Table 1). However, EBV viral loads in BENTA patients are not nearly as high as seen in chronic active EBV (CA-EBV) patients and other PIDs (46). These data suggest that EBV-specific immunity is impaired in BENTA patients, but pales in comparison to other PIDs such as X-linked lymphoproliferative syndrome (XLP) or MAGT1/CD27/CD70/ITK or Coronin1A deficiency diseases featuring exquisite susceptibility to severe EBV infection and disease (5, 20–22). In the next section, we speculate on why gain-of-function CARD11 mutations might confer susceptibility to moderate EBV viremia in BENTA disease.

## Mechanisms Underlying Benta Susceptibility to EBV

### Too Many B Cells, Too Few T/NK Cells?

The consequences of distorted antigen receptor signaling in the presence of GOF CARD11 mutations reverberate throughout the lymphoid lineage in BENTA patients. Indeed, the size and makeup of lymphocyte compartments may influence EBV status in certain patients. Most notably, constitutive, canonical NF-κB activity induced by GOF CARD11 signaling in B cells drives excessive B cell accumulation in BENTA patients and may predispose them to malignant transformation as additional mutations are acquired over time. In fact, two patients in our cohort developed B-cell tumors in adulthood (P1 and P11), although neither was associated with EBV infection. Transgenic expression of a constitutively active form of IKKβ (caIKKβ) promotes the survival of mature murine B cells *in vivo*, though it is not sufficient to induce lymphomagenesis (47). Indeed, NF-κB-induced tumor suppressor genes such as A20 and IκB provide important negative feedback on NF-κB signaling, which must be overcome to promote lymphomagenesis (48). This negative feedback remains intact in primary BENTA B cells and may explain why only a fraction of BENTA B cells exhibit p65 nuclear localization at any given time (23).

Epstein–Barr virus itself is not likely a contributing factor for B-cell lymphocytosis in BENTA, as EBV-negative patients also have high B cell numbers. Although NF-κB actively represses lytic infection (49), the proliferation of latently infected EBV^+^ B cells relies on NF-κB signaling through viral proteins such as LMP1 and LMP2A, which mimic CD40 and BCR signaling, respectively (5). Hence, constitutive NF-κB activity in BENTA patient B cells could better enable EBV to expand the pool of latently infected B cells. Perhaps this could manifest in increased viral reactivation and viremia as CARD11-dependent NF-κB activity oscillates in infected BENTA B cells. Regardless, the presence of EBV may increase the risk of B cell malignancy later in life, simply given the increased size of target B cell compartment. An expanded pool of naïve B cells may simply support an increased level of lytic infection at any given time in EBV-infected BENTA patients, contributing to consistently higher viral loads. In support of this notion, there is an unconfirmed case of EBV-driven Hodgkin lymphoma in the maternal grandfather of patient P16.

Could relative reductions in the T and NK cell compartments also compromise immunity to EBV? In addition to the critical role served by CD8^+^ T cells, CD4^+^ T cells, NK cells, and NKT cells are also implicated in clearing EBV infection (16, 17, 19). As shown in Table 1, the absolute number of CD8^+^ T cells and the ratio of CD4^+^/CD8^+^ T cells are within normal range in most patients reported thus far. Nevertheless, the low number of NK/NKT cells observed in certain patients (e.g., P14, P15, and P16) could contribute to persistent EBV viremia in those individuals. Patient P5 also has a lower percentage and absolute number of NK cells, presenting a potential culprit for her frequent susceptibility to other viral infections.

### Impaired T/NK Cell Function

A more likely explanation for uncontrolled EBV infection concerns impaired T cell function described in BENTA disease. *In vitro*, we observed poor proliferation and reduced IL-2 secretion from BENTA patient T cells stimulated with anti-CD3/anti-CD28 antibodies. This “anergic” response correlated with defects in TCR-mediated MAPK signaling and Ca^++^ flux (23, 35). Although the biochemical mechanisms remain nebulous, these defects are almost certainly linked to constitutive canonical NF-κB activation induced by GOF CARD11 signaling. Indeed, Krishna et al showed that restricting expression of constitutively active IKKβ (caIKKβ) to murine T cells also rendered them hyporesponsive to TCR/CD28 stimulation, marked by proximal TCR signaling defects and attenuated responses to bacterial infection (50). The authors connected some of these defects to enhanced expression of the transcriptional repressor Blimp-1, which has been shown to promote T cell exhaustion. Although we have not measured Blimp-1 in BENTA T cells, we recently characterized a profound, intrinsic defect in patient B cell differentiation linked to failed induction of Blimp-1 (41). Clearly much more work is required to understand how elevated NF-κB activation perturbs seemingly independent pathways downstream of TCR signaling. Regardless, it is tempting to speculate that any flaw in PLCγ1-mediated Ca^++^ flux underlies poor T cell-dependent control of EBV, as observed in more dramatic fashion in both ITK and MAGT1 deficiency (51–55).

Inactivation of CARD11 has also been shown to inhibit NK cell development and function (56). Although it is not clear how GOF CARD11 signaling may affect NK cells in BENTA, poor IL-2 secretion by anergic T cells upon stimulation could certainly weaken the NK cell response to viral infection. As mentioned earlier, some patients also display lower frequencies of NK and NKT cells, although EBV viremia is observed in several patients with normal NK/NKT counts. Thus, the increased susceptibility of BENTA patients to EBV could be linked to both excessive polyclonal B cell lymphocytosis and hyporesponsive T cells/NK cells that help in combating the viral infection.

### Dysregulation of Key Receptor–Ligand Signals Required for EBV Control

**Figure 1:**
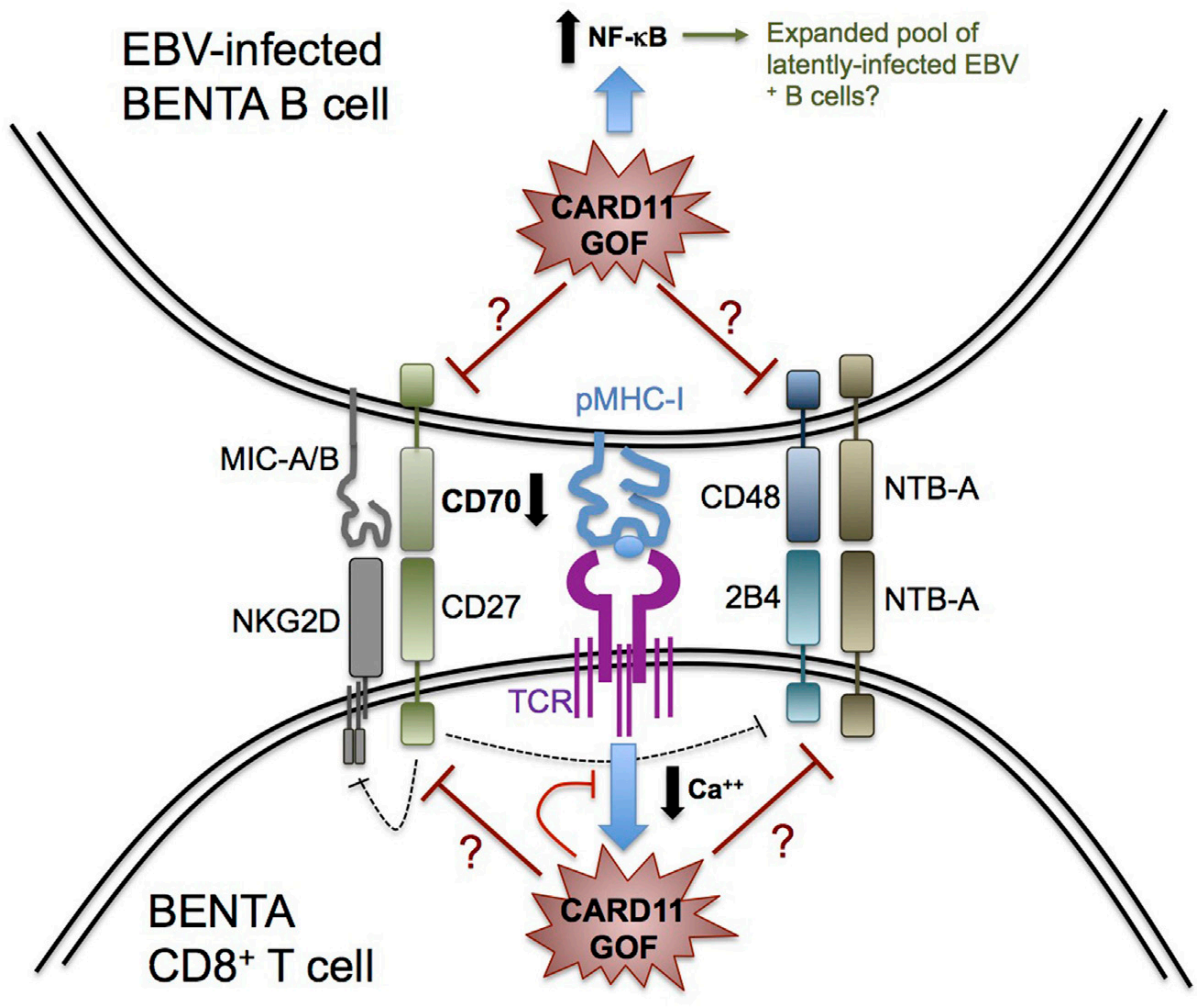
Possible determinants of impaired Epstein–Barr virus (EBV) control in *B*-cell *e*xpansion with *N*F-κB and *T*-cell *a*nergy (BENTA) disease. Schematic diagram summarizing key receptor–ligand interactions that govern CD8^+^ T cell recognition and killing of EBV-infected B cells, based on our knowledge of primary immune deficiencies featuring enhanced susceptibility to EBV-driven disease. CARD11 GOF signaling could perturb several molecular signals required for optimal cytolysis of EBV-infected B cells, including signaling lymphocyte activation molecule receptors (2B4, NTB-A), NKG2D, and CD27. For example, decreased CD70 expression on BENTA B cells could impair CD27 signaling and contribute to reduced NKG2D or 2B4 expression on BENTA T cells. Alternatively, attenuated TCR signaling (e.g., reduced Ca^++^ flux) likely contributes to BENTA patient T cell hyporesponsiveness, which could disrupt generation of CD8^+^ effector T cells with optimal cytotoxic function. Finally, elevated NF-κB signaling in B cells could accelerate the expansion of latently infected EBV^+^ B cells, contributing to detectable viremia as the virus continuously reactivates.

The removal of latently infected B cells by cytotoxic T and NK cells requires several receptor–ligand interactions for recognition, cell–cell conjugation, and cytolysis, some of which may be weakened in BENTA patients (Figure 1). For example, cognate engagement of EBV-infected B cells by T cells requires SAP-dependent signaling through two receptors belonging to the SLAM family: 2B4 and NTB-A (57–60). Whereas most SLAM receptors participate in homotypic interactions in *trans*, 2B4 recognizes a distinct ligand on target cells known as CD48. The expression of both NTB-A and CD48 is dramatically upregulated on the B cell surface upon EBV infection, which promotes T cell and NK cell activation during asymptomatic infection and acute infection, respectively (5, 61). Recruitment of SAP, a small SH2 adaptor protein, to the cytoplasmic tails of NTB-A and 2B4 upon ligand binding is required for conveying downstream signals that ensure strong T:B cell conjugation and B cell killing (62). In this regard, SAP deficiency in XLP-1 patients makes them exquisitely susceptible to severe, uncontrolled EBV infection due to debilitated 2B4 and NTB-A signaling, presenting as FIM/HLH (63, 64). Based on our published RNA-Seq data, BENTA B cells activated *in vitro* with polyclonal stimuli display normal expression of CD48 and NTB-A compared with healthy human donors (41). Whether perturbed 2B4 and/or NTB-A signaling in BENTA patient T cells may influence EBV predisposition remains unclear, but warrants further investigation.

Upon activation, the C-type lectin-like receptor NKG2D is also expressed on NK cells and CD8^+^ T cells and plays a major role in cytotoxic elimination of transformed and virally infected cells (65). The importance of NKG2D in EBV immunity was recently revealed by the discovery of X-linked immunodeficiency with magnesium defect, EBV infection, and neoplasia (XMEN) disease, caused by deficiency of the magnesium transporter MAGT1. Although NK cell and CD8^+^ T cell numbers are normal in XMEN patients, reduced intracellular Mg^++^ abrogates NKG2D receptor expression on activated NK cells and CD8^+^ T cells, which compromises cytolytic responses against EBV^+^ B cells (51, 66–68). Similarly, GOF CARD11 signaling may diminish NKG2D expression on BENTA NK cells or CD8^+^ T cells, perhaps linked to a Ca^++^ flux defect similar to that noted in XMEN T cells (54). On the other hand, there is no evidence for transcriptional upregulation of NKG2D receptor ligands MICA, MICB, and ULBPs on activated BENTA B cells compared with healthy control B cells. Simultaneous engagement of SAP-dependent 2B4 and SAP-independent NKG2D signaling is indispensable for CD8^+^ T cell-mediated killing of EBV-infected B cells (69), explaining why neither receptor (NKG2D or 2B4) can compensate for the absence of the other to maintain normal CTL activity in XMEN and XLP-1 patients. If constitutive CARD11 signaling indirectly impedes NKG2D or 2B4 signaling in BENTA CD8^+^ T cells/NK cells, this could jeopardize their ability to completely control EBV infection.

CD27, a costimulatory molecule belonging to the tumor necrosis factor receptor superfamily, is constitutively expressed on memory B cells and most T cells. CD27 engagement in B cells is known to play a key role in B cell activation and immunoglobulin synthesis (70). Our recent *in vitro* studies with BENTA B cells revealed an intrinsic defect in plasma cell differentiation and antibody production that correlated with poor induction of several genes related to plasma cell commitment, including CD27 (41), although CD27 expression is readily detected on patient T cells (data not shown). CD27 interacts with the ligand CD70, expressed transiently on activated B cells, T cells, and dendritic cells. EBV infection upregulates CD70 expression to greater levels on B cells (20). Recently described human patients with CD27 or CD70 deficiency present with similar disease phenotypes, including hypogammaglobulinemia, reduced memory B cells, increased viral infection, and EBV-induced lymphoproliferation and lymphoma. Heightened susceptibility to EBV-driven disease in these patients, despite normal numbers of T and NK cells, highlights a critical, non-redundant role for CD27–CD70 interactions in driving Ab responses and ensuring optimal cellular control of EBV (44, 71–74). Intriguingly, we recently discovered a significant reduction in CD70 expression on activated BENTA B cells *in vitro* compared with healthy control B cells (data not shown). Thereby, an impaired CD27–CD70 signaling axis in BENTA could significantly contribute to both specific Ab deficiency and impaired priming and function of EBV-specific CD8^+^ T cells. The latter could also be related to decreased NKG2D and 2B4 expression on memory CD8^+^ T cells, similar to CD70-deficient patients (44). Further exploration of a potential CD27-CD70 signaling deficit in BENTA patients is therefore warranted to elucidate a plausible mechanism to explain the inability of BENTA T and NK cells to fully contain EBV.

## Clinical Management of EBV in Benta Patients

Assuming B cell lymphocytosis may predispose BENTA patients to greater risk of B cell malignancy later in life, patients are monitored closely for any evidence of B cell clonal outgrowth, using flow cytometry and Ig heavy chain rearrangement analysis. EBV viral load is also measured regularly, as increases in detectable viremia may reflect further debilitation of CD8^+^ T cell and NK cell function and could theoretically contribute to B cell transformation. However, viral loads in most EBV^+^ BENTA patients remain comparatively low relative to CA-EBV and other PIDs (46).

To the best of our knowledge, only one patient (P6) was actively treated for EBV-related complications (35). This patient was hospitalized at age 4 with acute EBV infection, featuring profound adenopathy and splenomegaly, as well as immune thrombocytic purpura. Lymph node biopsies revealed substantial polyclonal B cell accumulation in follicular and parafollicular areas, mixed with moderate numbers of CD8^+^ and CD4^+^ T cells. At this time, years before the causative *CARD11* mutation was discovered, the patient was treated aggressively with intravenous immunoglobulin, rituximab, corticosteroids, and acyclovir. Symptoms resolved with treatment, and plasma EBV load was rendered undetectable by PCR. CD4^+^/CD8^+^ T cell ratio, which had dropped significantly during acute EBV infection, also recovered once infection was cleared. Following elective splenectomy 3 years later, his circulating B cell, T cell, and NK cell counts increased dramatically. This phenomenon has been observed in other patients following spleen removal (40) and likely reflects the loss of an important secondary lymphoid tissue niche for excess lymphocytes. Nevertheless, B cell counts in this patient remained 5–10 times higher than those noted in other BENTA patients. To control lymphocytosis, P6 was treated with methotrexate for 4 years until his lymphocyte count was reduced below 80 × 10^3^/μl (35).

This distinctive case provides an illustrative example of successful treatment for acute EBV infection and may represent a blueprint for care if EBV viremia or lymphocytosis increases rapidly in any BENTA patient. Unlike CA-EBV patients, BENTA patients should not require more radical clinical interventions, such as hematopoietic stem cell transplantation or administration of autologous cytotoxic T cells to combat EBV infection (46). In the future, pharmacological inhibitors of NF-κB activation may be an attractive therapeutic tool for reducing B cell numbers in BENTA patients but must be approached with caution to avoid exacerbating underlying T/NK cell immunodeficiency. Inhibitors of MALT1 protease, which dampen canonical NF-κB activity without completely blocking it, may be a more attractive option and have recently yielded promising results for treatment of B cell lymphoma and autoimmune disease (75–78). Future clinical management will ultimately be guided by more basic research into possible aforementioned mechanisms that might explain impaired CTL and NK cell function and compromised EBV control in BENTA disease.

## Conclusion

Although the current cohort of patients remains small, impaired control of EBV infection has emerged as a recurring problem in BENTA disease. In contrast to PIDs involving severe EBV-related complications (e.g., fulminant hepatitis and HLH) and complete deficiency of aforementioned receptors and signaling proteins required for EBV immunity, lower viremia in BENTA patients likely reflects attenuation, but not complete abrogation, of T and NK cell functions (Figure 1). Further research is required to connect CARD11 GOF signaling mechanistically to these moderate functional defects, which may involve aberrant signaling through NF-κB as well as other downstream signaling nodes, including JNK and mTORC1. Indeed, severe atopic disease observed in patients carrying CARD11 loss-of-function mutations can be attributed to defects in both NF-κB and mTORC1 activation in T cells (79), although none of these patients have presented with significant EBV infections. Continued identification and careful characterization of additional patients harboring novel CARD11 variants should yield further insights into how CARD11 signaling ultimately governs the immune response against EBV.

## Author Contributions

SA wrote the manuscript. PA assembled data for Table 1. HS edited the manuscript and provided clinical perspective. AS supervised the project and edited the manuscript.

## Conflict of Interest Statement

The authors declare that the research was conducted in the absence of any commercial or financial relationships that could be construed as a potential conflict of interest. The handling editor declared a shared affiliation, although no other collaboration, with the authors PA and HS.
